# 1203. Impact of Antimicrobial Stewardship Interventions on Hospital-Onset *Clostridioides difficile* Infections

**DOI:** 10.1093/ofid/ofad500.1043

**Published:** 2023-11-27

**Authors:** Taesung Kwon, Caroline E Dillon, Madhuri Duggirala, Daniel Berk, Tyler Barbour, Swati Vasireddy, Bridget Yablonski, Mark A Shelly

**Affiliations:** Geisinger, Danville, Pennsylvania; Geisinger Medical Center, Danville, Pennsylvania; Geisinger, Danville, Pennsylvania; Geisinger Medical Center, Danville, Pennsylvania; Geisinger Medical Center, Danville, Pennsylvania; Geisinger Medical Center, Danville, Pennsylvania; Geisinger, Danville, Pennsylvania; Geisinger Health System, Danville, PA

## Abstract

**Background:**

We carried out a case control study to document the effect of antimicrobial stewardship interventions (ASI) on hospital-onset CDI.

Types of antimicrobial stewardship intervention and implementation rate

**Figure:**
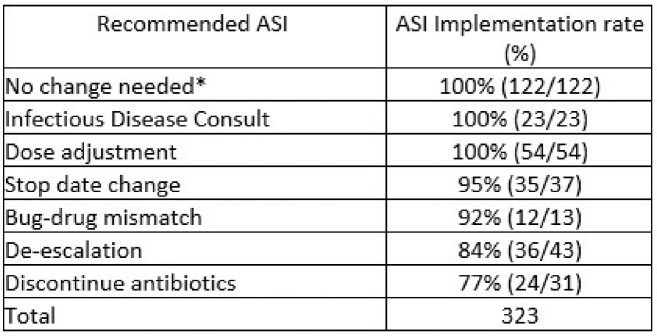

*Antimicrobial stewardship team reviews the case and recommends no change.

**Methods:**

We identified cases of laboratory-identified CDI at a medical school-affiliated medical center. Cases were identified by PCR as laboratory identified hospital onset CDI. Each case was matched with 2 cases where a stool PCR on the same hospital day was negative in the same month. Cases were limited to adults without a history of CDI in the last 3 months. Charts were reviewed for CDI risk factors, antibiotic exposure, and ASI documentation and follow-through.

**Results:**

From 2020 to 2021 there were 145 cases of HO-CDI, for which we assigned 125 cases with 250 in the matched referent group. A total of 323 ASIs were recorded in 184 charts. The ASI implementation rate was 87.2% with the lowest rate (77%) seen in “discontinue antibiotics” (Table). Of patients in the CDI group, 53.6% (67) had an ASI, compared to 47.2% (118) in the control (OR 1.29 (0.84 to 1.99), P = 0.25). Oftentimes (47.8%) these occurred on or after the day of the CDI test. Most risk factors for CDI were evenly distributed in both groups. Use of PPI/H2 blockers were seen more often in the cases (73.6% in cases vs 58.4% in controls, OR 2.00 (1.26 to 3.23), P = 0.004). The number of days with antibiotics and the cumulative number of antibiotic days was not different in patients tested for CDI. The median (IQR) of the number of days with antibiotic use in the 30 days prior to test was 6 (3.5 to 9) in the CDI and 7 (4 to 10) in the control. The total number of antibiotic days were 9 (5 to 14) in the CDI group and 10 (6 to 16) in the control group. No antibiotic class was seen more often in the CDI group. Macrolides, mostly azithromycin, were seen more often in the control group (OR 0.30 (0.09 to 0.82), P = 0.016).

**Conclusion:**

We did not see a difference in the rate of ASIs in patients who tested PCR positive or negative for CDI. The rate of ASI was lower than we anticipated, reducing the chance for ASI to show an effect. Most ASIs were followed when directions were given (87.2%), leaving too few not followed (17) to meaningfully assess differences. Diarrhea and CDI often occurred before the ASI was completed, reducing the opportunity for impact. To influence this outcome, ASI need to be implemented early in the antibiotic course to see an impact.

**Disclosures:**

**Mark A. Shelly, M.D.**, Rebiotrix: Advisor/Consultant

